# Social determinants of mental health in asthma: an exploratory study

**DOI:** 10.3389/falgy.2024.1464948

**Published:** 2025-02-05

**Authors:** Sarah A. Hiles, Hayley Lewthwaite, Vanessa L. Clark, Anne E. Vertigan, Amber Smith, Vanessa M. McDonald

**Affiliations:** ^1^ School of Psychological Sciences, University of Newcastle, Callaghan, NSW, Australia; ^2^ Asthma and Breathing Research Program, Hunter Medical Research Institute, New Lambton Heights, NSW, Australia; ^3^ Centre for Research Excellence in Treatable Traits, College of Health, Medicine, and Wellbeing, University of Newcastle, New Lambton Heights, NSW, Australia; ^4^ Speech Pathology Department, John Hunter Hospital, New Lambton Heights, NSW, Australia; ^5^ Department of Respiratory and Sleep Medicine, John Hunter Hospital, New Lambton Heights, NSW, Australia

**Keywords:** asthma, anxiety, depression, resilience, social determinants, sex, age, employment

## Abstract

**Introduction:**

Asthma and mental health problems co-occur at high rates. In context of a holistic approach to health, considering the extent to which social determinants relate to mental health in people with asthma helps identify health inequity and inform population-level preventative strategies. The aim of the current exploratory study was to examine how social determinants are associated with depression, anxiety and resilience in people with mild-moderate and severe asthma.

**Methods:**

A cross-sectional study of 144 adults (aged ≥18 years) with a diagnosis of asthma was conducted. Participants were classified as having mild-moderate asthma or severe asthma based on international guidelines. As part of a multidimensional assessment, participants self-reported age, sex, ethnicity, country of birth, living arrangements, employment, and postcode. They also completed validated self-report questionnaires for depression and anxiety [Hospital Anxiety and Depression Scale (HADS)], and resilience [Resilience Scale (RS-25)]. Bayesian regression analyses were conducted to examine the extent to which social determinants were associated with depression, anxiety and resilience.

**Results:**

74 participants had mild-moderate asthma and 70 participants had severe asthma. Participants were on average 60 years old (SD = 14), 72% were female, 94% were Caucasian, 94% were Australian-born, 26% lived alone, 42% were working full- or part-time, and 83% lived in a major city of Australia. Anxiety and depression were relatively common (35% anxiety; 16% depression using HADS threshold of scores ≥8). Few social determinants were associated with depression, anxiety and/or resilience. Older age was associated with greater resilience. Females had higher levels of anxiety compared to males. Compared to participants currently working full- or part-time, those who were not working or studying due to their health had worse depressive symptoms and those who were not working for other reasons such as retirement had greater resilience.

**Discussion:**

As in the general population, age, sex and employment/student status were associated with components of mental health in people with asthma. Although limited by the small sample size and sociodemographic homogeneity, the findings of this exploratory study contribute to the large body of work fostering a holistic approach to health and striving for health equity in people with asthma, particularly those who experience mental health problems.

## Introduction

Mental health is a crucial aspect of quality of life in asthma. Yet, depression and anxiety are 1.5–2.4 times more common in people with asthma than people without asthma (1, 2). In severe asthma—the most debilitating form of the disease—depression and anxiety are even more common (3–6). Depression and anxiety are associated with poorer asthma outcomes, including increased risk of exacerbation (6–11). Reciprocally related to depression and anxiety is the concept of resilience (12–14). Resilience involves the ability of “bounce back” after experiencing adversity and has been linked to improved health behaviors and outcomes across a range of health conditions (15–17). This concept has been infrequently examined in adults with asthma; however, studies in adolescents demonstrate asthma is associated with lower resilience (18, 19).

Multiple reasons likely explain the high prevalence of poor mental health in people with asthma. From a population perspective, one important set of reasons to consider is social determinants of health (20). The World Health Organization's Social Determinants of Health Framework highlights the critical influence of the social and economic environment on health, including biomedical factors, mental health, and health behavior (20, 21). Social position affects a person's living and working conditions, their susceptibility to illness and their experience of illness. Social determinants are relevant across all of society, since health inequity or disadvantage is thought to exist on a social gradient whereby those in a lower social position have poorer health than those who are higher (22). Positive and negative influences also accumulate across an individual's lifespan, with the effects of stress and illness mounting as people age (20, 22). The role of social determinants in asthma and mental health have been reviewed previously (20, 23–25). The current study seeks to bring these literatures together, focusing on the mental health of people with mild-moderate and severe asthma.

The aim of the current exploratory study was to examine how social determinants are associated with depression, anxiety and resilience in people with mild-moderate and severe asthma. We focused on socio-demographic determinants of age, sex, ethnicity, migrant status, living arrangements, employment status, and the neighborhood socio-economic disadvantage and geographical remoteness of participants’ residence. We hypothesized that younger age, male sex, Caucasian ethnicity, Australian birthplace, living with a partner, being employed, lower neighborhood socio-economic disadvantage and living in a major city of Australia would be associated with lower depression and anxiety symptoms and greater resilience.

## Methods

### Design

This study was a secondary analysis of data collected as part of a cross-sectional, observational study of adults with mild-moderate and severe asthma in Newcastle, Australia (ACTRN12620000283976)—Understanding Breathlessness in Asthma (26). The study was designed and reported according to STROBE guidelines for observational studies.

### Ethics

The Human Research Ethics Committee of the Hunter New England Local Health District (2019/ETH12515) approved the study protocol. All participants provided written informed consent prior to completing study assessments.

### Participants

Participants were recruited from the Department of Respiratory and Sleep Medicine's Ambulatory care clinics at the John Hunter Hospital, the research databases of the Department of Respiratory and Sleep Medicine and Hunter Medical Research Institute, and through general and social media advertisement.

Participants were eligible to participate if they were aged ≥18 years, with a physician confirmed diagnosis of asthma and objective evidence of variable airflow limitation within the past 10 years or assessed as part of the study. Variable airflow limitation was defined as: a change in forced expiratory volume in one second (FEV_1_) of ≥200 ml or ≥12% 15 min following bronchodilator administration and/or airway hyper-responsiveness (fall in FEV_1_ ≥15%) in response to hypertonic saline challenge and/or peak flow diurnal variation of ≥15% or >50 ml.

Eligible participants were considered to have severe asthma if they met international guideline criteria (27). That is, to control asthma, they required: (1) high-dose inhaled corticosteroids (>1,000 μg beclomethasone equivalent) and a second controller (may include long-acting beta agonists, and/or long-acting muscarinic antagonists, and/or maintenance oral corticosteroids ≥50% of the past year, and/or montelukast, and/or theophylline); or (2) monoclonal antibody (mAb) therapy for asthma; or their asthma remained uncontrolled despite such therapy, defined as:

• Asthma Control Questionnaire-7 (ACQ-7) score ≥1.5, and/or
• Frequent severe (≥2 systemic corticosteroids of ≥3 days each) and/or a serious (hospitalization, intensive care unit stay, mechanical ventilation) exacerbation in the previous 12 months, and/or
• Persistent airflow limitation [pre-bronchodilator FEV_1_ to forced vital capacity (FVC) ratio of <0.7 and FEV_1_ <80% predicted].

Criteria for study exclusion were: (1) currently pregnant; (2) high dependence on medical care, including recent intensive care unit admission; (3) significant life-limiting comorbidity; (4) current lung cancer or other blood, lymphatic or solid organ malignancy; (5) a primary diagnosis of lung disease other than asthma; or (6) cognitive impairment, poor English language skills or significant untreated hearing impairment that prevented completion of data collection forms or understanding of verbal instructions. Participants who were otherwise eligible but had experienced an asthma exacerbation (use of oral corticosteroids or an increase from maintenance dose of oral corticosteroids for ≥3 days) in the past four weeks were included but had their assessment visit postponed for four weeks.

### Procedure

Participants completed a comprehensive, multidimensional assessment across two in-person assessment visits, approximately two weeks apart. Participants were also provided with a questionnaire pack to take home and complete between visits. The assessment included measures of sociodemographic characteristics, pulmonary function, asthma control, mental health, breathlessness, comorbidity, exercise capacity, and physical activity. Measures relevant to the current study are described below.

### Asthma assessment

For assessment of airflow limitation and reversibility, spirometry was completed (Medgraphics, CPFS/D USB Spirometer, BreezeSuite v8.5-v8.6, MGC Diagnostics) pre- and 15 min post-administration of 400 μg of salbutamol and 42 μg of ipratropium. Testing was performed according to international ATS/ERS standards, including withholding of bronchodilator medications for recommended time periods (28). The Global Lung Initiative normative reference set was used to calculate percent of predicted values (29). To determine asthma severity, a trained researcher conducted a structured interview, identifying asthma medications and healthcare utilisation over the past 12 months, and the presence of a diagnosis of severe asthma.

### Independent variables

Participants self-reported the following data that were used to define the social determinants used for analysis: date of birth, sex (male, female, intersex), ethnicity, country of birth, living arrangements (*a priori* defined categories: lives alone, in a share house, with a spouse/partner, with family, and in another arrangement), employment (*a priori* categories: working full- or part-time, studying, seeking work, domestic duties, not studying or working due to their health, not working for other reasons/retired), and postcode and suburb of their home address. Date of birth was used to calculate age at enrolment in the study. Postcode was linked to the Index of Relative Socio-economic Disadvantage (IRSD), an index from the Australian Socio-Economic Indexes for Areas (30). The IRSD is a relative score that summarizes the economic and social conditions of individuals and households in the area where the participant lives, based on Australian census data such as income, education, employment, occupations, housing and family structure. Lower scores indicate greater neighborhood disadvantage, with the median score 1002 [interquartile range (IQR) 957–1,047]. It is not indicative of the socioeconomic disadvantage of the individual themselves. Suburb was searched in the 2021 Australian Statistical Geography Standard (ASGS) Edition 3 (31, 32) to define whether the participant's home location was in one of five classifications: a major city of Australia, inner regional Australia, outer regional of Australia, remote Australia or very remote Australia. These categories summarize residents' relative access to services.

### Dependent variables

Depression and anxiety were measured using the two subscales of the Hospital Anxiety and Depression Scale (HADS) (33). Each subscale ranges from 0 to 21, with higher scores indicating worse symptoms. Scores ≥8 on each subscale are indicative of possible clinically important symptoms of depression or anxiety. Resilience was measured using the 25-item Resilience Scale (RS-25) (34). The scale ranges from 25 to 175, with higher scores indicating greater resilience. Wagnild (35) suggests that scores over 145 indicate moderately high to high resilience.

### Statistical analysis

Analyses were conducted in Stata BE 17.0 (StataCorp LLC). Descriptive statistics were calculated for social determinants and mental health variables. Bayesian linear regressions were conducted to determine the relationship between each social determinant and depression, anxiety, or resilience, controlling for asthma diagnosis (mild-moderate vs. severe), using the default settings [predictor priors: normal (0,10000); MCMC iterations: 12,500; burn-in 2,500]. We tested whether including an interaction between the social determinant and asthma diagnosis improved model fit using log marginal likelihood. In all instances, the model without an interaction had the largest marginal likelihood, so these were reported. Categories with fewer than 5 participants were excluded from regression analyses. Centrality of effect sizes were reported as mean with 95% credible intervals using the highest density interval. The associations were not adjusted for confounding, as the aim was to provide measures of total effect.

## Results

### Characterizing social determinants and mental health in mild-moderate and severe asthma

There were 74 participants with mild-moderate asthma and 70 with severe asthma. Participants were on average 60 years old (SD = 14), 72% were female, 94% were Caucasian, 94% were Australian-born, 26% lived alone, 42% were working full- or part-time, and 83% lived in a major city of Australia (Table 1). Six participants (4%) were Aboriginal or Torres Strait Islander. Values on the index of relative economic social disadvantage were similar to the national median of 1,002 (30). Overall, the sample had similar or better social advantage than the general Australian population: 3.8% of Australians are First Nations Aboriginal or Torres Strait Islander (36), 30.7% were born overseas (37), 0.5% are homeless (38), 4.0% are unemployed (39), 13.4% live below the poverty line of 50% of median income (40), and 10.1% live in outer regional, remote or very remote locations (41). Social determinants were similar in value for mild-moderate and severe asthma, although the severe asthma group had more individuals who were males, fewer working full- or part-time, and more living in inner regional Australia.

**Table 1:** Descriptive statistics for **s**ocial determinants and mental health in participants with mild-moderate and severe asthma.

		Total (*N* = 144)	Mild-moderate asthma (*N* = 74)	Severe asthma (*N* = 70)
Social determinants				
Age (years), mean (SD)		59.7 (13.5)	58.2 (13.8)	61.3 (13.2)
Sex, *N* (%)	Male	41 (28.5)	17 (23.0)	24 (34.3)
	Female	103 (71.5)	57 (77.0)	46 (65.7)
Ethnicity, *N* (%)	Caucasian	135 (93.8)	68 (91.9)	67 (95.7)
	Not Caucasian	9 (6.3)	6 (8.1)	3 (4.3)
Migrant status, *N* (%)	Australian-born	134 (93.7)	69 (93.2)	65 (94.2)
	Overseas-born	9 (6.3)	5 (6.8)	4 (5.8)
Living arrangements, *N* (%)	Living alone	38 (26.4)	19 (25.7)	19 (27.1)
	Living in a share house	2 (1.4)	1 (1.4)	1 (1.4)
	Living with a spouse/partner	57 (39.6)	32 (43.2)	25 (35.7)
	Living with family	46 (31.9)	22 (29.7)	24 (34.3)
	Other arrangement	1 (0.7)	0 (0.0)	1 (1.4)
Employment status, *N* (%)	Studying	3 (2.1)	1 (1.4)	2 (2.9)
	Seeking work	1 (0.7)	0 (0.0)	1 (1.4)
	Working full- or part-time	60 (41.7)	39 (52.7)	21 (30.0)
	Domestic duties	3 (2.1)	2 (2.7)	1 (1.4)
	Not studying or working due to health	19 (13.2)	6 (8.1)	13 (18.6)
	Not working for other reasons/retired	58 (40.3)	26 (35.1)	32 (45.7)
Neighborhood socio-economic disadvantage, mean (SD)	Index of relative economic disadvantage	994.3 (37.1)	1,002.5 (30.2)	985.7 (41.8)
Geographical remoteness, *N* (%)	Major city of Australia	119 (82.6)	65 (87.8)	54 (77.1)
	Inner regional Australia	24 (16.7)	9 (12.2)	15 (21.4)
	Outer regional Australia	1 (0.7)	0 (0.0)	1 (1.4)
Mental health				
Depression (HADS), median (Q1, Q3)		3.5 (2.0, 6.0)	3.5 (1.0, 6.0)	3.5 (2.0, 7.0)
Possible clinically important depression (HADS depression score ≥8), *N* (%)		23 (16.4)	11 (15.7)	12 (17.1)
Anxiety (HADS), mean (SD)		6.4 (3.9)	6.6 (4.1)	6.1 (3.7)
Possible clinically important anxiety (HADS anxiety score ≥8), *N* (%)		49 (35.0)	29 (41.4)	20 (28.6)
Resilience (RS-25), mean (SD)		141.4 (20.6)	142.6 (19.2)	140.2 (22.0)
Moderately high-high resilience (RS-25 score >145), *N* (%)		72 (52.1)	37 (53.6)	35 (50.7)

HADS, Hospital Anxiety and Depression Scale; 25th percentile, Q1; 75th percentile, Q3; RS-25, Resilience scale; SD, standard deviation.

High levels of anxiety and depression were common (Table 2). Using a HADS threshold of ≥8, 16% had clinically significant levels of depressive symptoms and 35% had clinically significant levels of anxiety symptoms. Mean resilience was 141.4 (SD = 20.6), which is a similar value to studies across various populations reviewed by Wagnild (35). Mental health variables were of similar magnitude between mild-moderate asthma and severe asthma.

**Table 2:** Social determinants predicting mental health related variables, controlling for severe asthma diagnosis.

Social determinants		Log depression	Anxiety	Resilience
		Posterior mean of β (95% credible interval)	Posterior mean of β (95% credible interval)	Posterior mean of β (95% credible interval)
Age (years)		0.006 (−0.003, 0.015)	−0.05 (−0.10, 0.00)	**0.27** (**0.02, 0.53)**
Sex	Male	Reference	Reference	Reference
	Female	0.095 (−0.154, 0.359)	**1.93** (**0.59, 3.37)**	−2.18 (−9.88, 5.52)
Ethnicity	Caucasian	Reference	Reference	Reference
	Not Caucasian	0.113 (−0.365, 0.618)	1.62 (−0.96, 4.23)	1.79 (−13.14, 16.55)
Migrant	Australian-born	Reference	Reference	Reference
	Overseas-born	−0.347 (−0.817, 0.153)	−0.59 (−3.09, 1.84)	−0.18 (−13.91, 13.43)
Living arrangements^a^	Living alone	Reference	Reference	Reference
	Living with a spouse/partner	−0.074 (−0.369, 0.239)	0.26 (−1.48, 1.98)	3.07 (−5.49, 11.94)
	Living with family	−0.024 (−0.347, 0.283)	0.68 (−0.90, 2.38)	−2.34 (−11.58, 7.07)
Employment status^b^	Working full- or part-time	Reference	Reference	Reference
	Not studying or working due to health	**0.516** (**0.131, 0.904)**	0.90 (−1.12, 2.89)	−0.61 (−11.28, 10.51)
	Not working for other reasons/retired	0.009 (−0.267, 0.285)	−0.90 (−2.39, 0.51)	**9.98** (**2.39, 17.77)**
Neighborhood socio-economic disadvantage	Index of relative economic disadvantage	−0.002 (−0.005, 0.002)	−0.02 (−0.03, 0.00)	0.10 (−0.02, 0.21)
Geographical remoteness^c^	Major city of Australia	Reference	Reference	Reference
	Inner regional Australia	0.149 (−0.190, 0.478)	0.53 (−1.25, 2.39)	−2.11 (−11.71, 7.85)

Reference categories are denoted with the word “Reference”. Analyses where the 95% credible interval does not include 0 are bolded.

^a^ Categories of living in a share house and other arrangements were excluded due to small cell size.

^b^ Categories of studying and domestic studies were excluded due to small cell size.

^c^ Category of outer regional Australia was excluded due to small cell size.

### Social determinants as predictors of depression, anxiety and resilience

Overall, few social determinants were associated with mental health (Table 2). Older age was associated with greater resilience. Females had greater levels of anxiety compared to males. Compared to participants currently working full- or part-time, those who were not working or studying due to their health had worse depressive symptoms and those who were not working for other reasons such as retirement had greater resilience.

## Discussion

Quantifying the relationship between social determinants and mental health among people with asthma contributes to a holistic approach to health in this population. We see that age, sex and employment may be key social drivers of mental health in people with asthma. However, the reliability and generalizability of these findings are limited by the small and relatively homogenous cohort, which was biased toward better social advantage and low geographic dispersion compared with the general Australian population. Indeed, although some social determinants were associated with depression, anxiety and/or resilience, the pattern across these mental health measures was inconsistent. Person-level health or psychological determinants such as disability and interpersonal relationships may be more strongly associated with mental health at an individual-level, whereas social determinants may be observable in large-scale studies and important at a population-level.

Age and sex are often considered important social determinants of mental health, although the strength and directions of the associations vary in different contexts. In the current study, older age was associated with greater resilience. The literature is mixed in this regard, with reports of lower resilience with advancing age, likely due to cumulative life stressors or homeostatic mechanistic changes (42, 43), and other studies demonstrating high resilience amongst the oldest-old (44, 45). A review of the RS-25 indicated no age-related differences in mean resilience scores (35). Our observed association between increasing age and resilience may also partly explain or be explained by our finding that greater resilience was associated with not working for reasons such as retirement. Wellbeing tends to be maintained or even improve after retirement (46). The observation that female sex was associated with greater levels of anxiety is consistent with previous research in the general population (47). Although some studies associate female sex or gender with depression, other data indicate the gender gap in depression is narrowing over time (48) and the gap could be a consequence of how symptoms are assessed (49). We acknowledge the critical limitation that we asked participants for their sex, which is biological in nature, rather than gender, which is social in nature. Thus, we did not capture if participants were transgender, non-binary or had another self-description of their gender.

Not working due to health was associated with depression. This association is likely bidirectional. Depression may increase the risk of unemployment or underemployment, either directly or indirectly through another factor such as disability (50, 51). Depression is associated with greater levels of disability in chronic disease, and greater disability leads to lower likelihood of working. It also affects performance at work; our previous research indicates that depression and anxiety are associated with presenteeism (working suboptimally due to health) in people with severe asthma, adjusting for asthma control (52). Economic and social consequences of unemployment, underemployment, absenteeism or presenteeism, such as lost earnings, arrested career progression, and poverty may also contribute to depression. The current study did not capture whether it was asthma or another physical or mental health problem that caused participants to not work, but this would be an important differentiation to understand this association further.

Ethnicity and migrant status, living arrangements, socio-economic disadvantage and geographic remoteness were not associated with any of the mental health variables in this study, and yet their true effect on an individual or the population may be profound. Most of our sample were Caucasian, Australian-born, and recruited from one geographic region, so our ability to detect the effect of these variables on mental health was limited. Living arrangement does not capture the quality of social relationships, which is likely to be a more influential driver of mental health. Socio-economic disadvantage and remoteness were characterized in terms of an individual's address, rather than an individual's own advantage, income, or access to healthcare or social services. Therefore, these variables likely underestimated the true effects of these social determinants on mental health.

We saw little difference in social determinants in severe compared with mild-moderate asthma, which is consistent with a previous study of a large pediatric sample that showed a similar lack of difference (53). Alachraf and colleagues (53) found that although race/ethnicity, insurance coverage and parental education attainment were associated with emergency department visits in children with asthma, the strength of association did not differ according to severity of asthma. Social determinants frameworks would suggest that more severe disease should lead to greater accumulated disadvantage across a lifespan (20, 22), therefore, examining age-disease interactions in larger cohorts is warranted.

The key strength of this study was that the cohort was enriched with a large sample of individuals with severe asthma, who are typically underrepresented in asthma research. This has allowed us to characterize social determinants of mental health in this important group. Regarding study limitations, this was a cross-sectional observational study investigating simple and potentially confounded effects, so no inference on causality can be made with these data. Several potentially important socio-demographic social determinants were not captured in this study; in particular, we had no direct measures of household income or poverty, education, housing security or social inclusion. Furthermore, since participants were a convenience sample, some socially disadvantaged groups were underrepresented, which introduced bias in the sample. In particular, migrants from developing countries, homeless, job-seekers, persons with low income and rural or remote located Australians were poorly captured in this study. These groups tend to have poorer health status.

Our exploratory findings contribute to the large body of work fostering a holistic approach to health and striving for health equity in people with asthma, particularly those who experience mental health problems. Larger studies that overcome sampling limitations will help provide stronger, more actionable evidence. Screening for and intervening on social determinants of health improves outcomes for asthma and mental health (23, 54, 55). Based on our limited findings, supporting the mental health and resilience of younger people, females and those not working due to their health is paramount. These groups may be the target of population-levels interventions that aim to reduce health inequity and support mental health in people with asthma.

## Data Availability

The raw data supporting the conclusions of this article will be made available by the authors, without undue reservation.

## References

[1] Scott KM Von Korff M Ormel J Zhang M-y Bruffaerts R Alonso J Mental disorders among adults with asthma: results from the world mental health survey. Gen Hosp Psychiatry. (2007) 29(2):123–33. 10.1016/j.genhosppsych.2006.12.006 17336661 PMC1913936

[2] Strine TW Mokdad AH Balluz LS Gonzalez O Crider R Berry JT Depression and anxiety in the United States: findings from the 2006 behavioral risk factor surveillance system. Psychiatr Serv. (2008) 59(12):1383–90. 10.1176/ps.2008.59.12.1383 19033164

[3] Amelink M Hashimoto S Spinhoven P Pasma HR Sterk PJ Bel EH Anxiety, depression and personality traits in severe, prednisone-dependent asthma. Respir Med. (2014) 108(3):438–44. 10.1016/j.rmed.2013.12.012 24462260

[4] Shaw DE Sousa AR Fowler SJ Fleming LJ Roberts G Corfield J Clinical and inflammatory characteristics of the European U-BIOPRED adult severe asthma cohort. Eur Respir J. (2015) 46(5):1308–21. 10.1183/13993003.00779-2015 26357963

[5] Carvalho N Ribeiro P Ribeiro M Nunes M Cukier A Stelmach R. Asma e doença pulmonar obstrutiva crônica: uma comparação entre variáveis de ansiedade e depressão. J Bras Pneumol. (2007) 33(1):1–6. 10.1590/S1806-37132007000100004 17568861

[6] McDonald VM Hiles SA Godbout K Harvey ES Marks GB Hew M Treatable traits can be identified in a severe asthma registry and predict future exacerbations. Respirology. (2019) 24(1):37–47. 10.1111/resp.13389 30230137

[7] McCauley E Katon W Russo J Richardson L Lozano P. Impact of anxiety and depression on functional impairment in adolescents with asthma. Gen Hosp Psychiatry. (2007) 29(3):214–22. 10.1016/j.genhosppsych.2007.02.003 17484938 PMC2770903

[8] Schneider A Löwe B Meyer FJ Biessecker K Joos S Szecsenyi J. Depression and panic disorder as predictors of health outcomes for patients with asthma in primary care. Respir Med. (2008) 102(3):359–66. 10.1016/j.rmed.2007.10.016 18061424

[9] Baiardini I Sicuro F Balbi F Canonica GW Braido F. Psychological aspects in asthma: do psychological factors affect asthma management? Asthma Res Pract. (2015) 1(1):7. 10.1186/s40733-015-0007-1 27965761 PMC5142316

[10] Deshmukh VM Toelle BG Usherwood T O'Grady B Jenkins CR. Anxiety, panic and adult asthma: a cognitive-behavioral perspective. Respir Med. (2007) 101(2):194–202. 10.1016/j.rmed.2006.05.005 16781132

[11] Roy-Byrne PP Davidson KW Kessler RC Asmundson GJG Goodwin RD Kubzansky L Anxiety disorders and comorbid medical illness. Gen Hosp Psychiatry. (2008) 30(3):208–25. 10.1016/j.genhosppsych.2007.12.006 18433653

[12] Wermelinger Ávila MP Lucchetti ALG Lucchetti G. Association between depression and resilience in older adults: a systematic review and meta-analysis. Int J Geriatr Psychiatry. (2017) 32(3):237–46. 10.1002/gps.4619 27805730

[13] Beutel ME Glaesmer H Wiltink J Marian H Brahler E. Life satisfaction, anxiety, depression and resilience across the life span of men. Aging Male. (2010) 13(1):32–9. 10.3109/13685530903296698 19842788

[14] Hu T Zhang D Wang J. A meta-analysis of the trait resilience and mental health. Pers Individ Dif. (2015) 76:18–27. 10.1016/j.paid.2014.11.039

[15] Jin Y Bhattarai M Kuo WC Bratzke LC. Relationship between resilience and self-care in people with chronic conditions: a systematic review and meta-analysis. J Clin Nurs. (2023) 32(9-10):2041–55. 10.1111/jocn.16258 35194870

[16] Stewart DE Yuen T. A systematic review of resilience in the physically ill. Psychosomatics. (2011) 52(3):199–209. 10.1016/j.psym.2011.01.036 21565591

[17] Cal SF Sá L Glustak ME Santiago MB. Resilience in chronic diseases: a systematic review. Cogent Psychology. (2015) 2(1):1024928. 10.1080/23311908.2015.1024928

[18] Ciprandi G Marseglia GL Licari A Castagnoli R Ciprandi R. Resilience is low in adolescents with asthma and independent of asthma control. Acta Biomed. (2022) 93(2):e2022054. 10.23750/abm.v93i2.11751 35546022 PMC9171869

[19] Hopkins KD Shepherd CC Taylor CL Zubrick SR. Relationships between psychosocial resilience and physical health status of Western Australian urban aboriginal youth. PLoS One. (2015) 10(12):e0145382. 10.1371/journal.pone.0145382 26716829 PMC4696679

[20] Alegría M NeMoyer A Falgàs Bagué I Wang Y Alvarez K. Social determinants of mental health: where we are and where we need to go. Curr Psychiatry Rep. (2018) 20(11):95. 10.1007/s11920-018-0969-9 30221308 PMC6181118

[21] Solar O Irwin A. A Conceptual Framework for Action on the Social Determinants of Health. Social Determinants of Health Discussion Paper 2 (Policy and Practice). Geneva: World Health Organization (2010).

[22] Marmot M Bell R. Social inequalities in health: a proper concern of epidemiology. Ann Epidemiol. (2016) 26(4):238–40. 10.1016/j.annepidem.2016.02.003 27084546

[23] Sullivan K Thakur N. Structural and social determinants of health in asthma in developed economies: a scoping review of literature published between 2014 and 2019. Curr Allergy Asthma Rep. (2020) 20(2):5. 10.1007/s11882-020-0899-6 32030507 PMC7005090

[24] Silva M Loureiro A Cardoso G. Social determinants of mental health: a review of the evidence. Eur J Psychiatry. (2016) 30(4):259–92. https://psycnet.apa.org/record/2017-05809-003

[25] Grant T Croce E Matsui EC. Asthma and the social determinants of health. Ann Allergy Asthma Immunol. (2022) 128(1):5–11. 10.1016/j.anai.2021.10.002 34673220 PMC8671352

[26] Lewthwaite H Gibson PG Guerrero PDU Smith A Clark VL Vertigan AE Understanding breathlessness burden and psychophysiological correlates in asthma. J Allergy Clin Immunol Pract. (2024) 12(10):2754–63.e17. 10.1016/j.jaip.2024.06.019 38906398

[27] Chung KF Wenzel SE Brozek JL Bush A Castro M Sterk PJ International ERS/ATS guidelines on definition, evaluation and treatment of severe asthma. Eur Respir J. (2014) 43(2):343–73. 10.1183/09031936.00202013 24337046

[28] Graham BL Steenbruggen I Miller MR Barjaktarevic IZ Cooper BG Hall GL Standardization of spirometry 2019 update. An official American thoracic society and European respiratory society technical statement. Am J Respir Crit Care Med. (2019) 200(8):e70–88. 10.1164/rccm.201908-1590ST 31613151 PMC6794117

[29] Quanjer PH Stanojevic S Cole TJ Baur X Hall GL Culver BH Multi-ethnic reference values for spirometry for the 3–95-yr age range: the global lung function 2012 equations. Eur Respir J. (2012) 40(6):1324–43. 10.1183/09031936.00080312 22743675 PMC3786581

[30] Australian Bureau of Statistics. Socio-Economic Indexes for Areas (SEIFA). Australia Canberra, Australia: Australian Bureau of Statistics (2023). Available online at: https://www.abs.gov.au/statistics/people/people-and-communities/socio-economic-indexes-areas-seifa-australia/latest-release#index-of-relative-socio-economic-advantage-and-disadvantage-irsad- (cited June 30, 2024)

[31] Australian Bureau of Statistics. Australian Statistical Geography Standard (ASGS) Edition 3 Canberra. Australia: Australian Bureau of Statistics (2021). Available online at: https://www.abs.gov.au/statistics/standards/australian-statistical-geography-standard-asgs-edition-3/latest-release (cited June 30, 2024)

[32] Australian Government Department of Health and Aged Care. Health Workforce Locator Canberra. Australia: Australian Government Department of Health and Aged Care (2024). Available online at: https://www.health.gov.au/resources/apps-and-tools/health-workforce-locator/app (cited June 30, 2024)

[33] Zigmond AS Snaith RP. The hospital anxiety and depression scale. Acta Psychiatr Scand. (1983) 67(6):361–70. 10.1111/j.1600-0447.1983.tb09716.x 6880820

[34] Wagnild GM Young HM. Development and psychometric evaluation of the resilience scale. J Nurs Meas. (1993) 1(2):165–78. 7850498

[35] Wagnild G. A review of the resilience scale. J Nurs Meas. (2009) 17(2):105–13. 10.1891/1061-3749.17.2.105 19711709

[36] Australian Bureau of Statistics. Estimates of Aboriginal and Torres Strait Islander Australians Canberra. Australia: Australian Bureau of Statistics (2023). Available online at: https://www.abs.gov.au/statistics/people/aboriginal-and-torres-strait-islander-peoples/estimates-aboriginal-and-torres-strait-islander-australians/latest-release#cite-window1 (cited June 30, 2024)

[37] Australian Bureau of Statistics. Australia’s Population by Country of Birth Canberra. Australia: Australian Bureau of Statistics (2024). Available online at: https://www.abs.gov.au/statistics/people/population/australias-population-country-birth/latest-release (cited June 30, 2024)

[38] Australian Bureau of Statistics. Estimating Homelessness: Census. Canberra: Australian Bureau of Statistics (2023). Available online at: https://www.abs.gov.au/statistics/people/housing/estimating-homelessness-census/latest-release (cited June 30, 2024)

[39] Australian Bureau of Statistics. Labour Force, Australia Canberra. Australia: Australian Bureau of Statistics (2024). Available online at: https://www.abs.gov.au/statistics/labour/employment-and-unemployment/labour-force-australia/latest-release (cited July 2, 2024)

[40] Davidson P Bradbury B Wong M. Poverty in Australia 2022: A Snapshot. Strawberry Hills, Australia: Australian Council of Social Service (ACOSS) and UNSW Sydney (2022).

[41] Australian Institute of Health and Welfare. Rural and Remote Health Canberra. Australia: Australian Institute of Health and Welfare, Australian Government (2024). Available online at: https://www.aihw.gov.au/reports/rural-remote-australians/rural-and-remote-health (cited June 30, 2024)

[42] Hadley EC Kuchel GA Newman AB Allore HG Bartley JM Bergeman CS Report: NIA workshop on measures of physiologic resiliencies in human aging. J Gerontol A. (2017) 72(7):980–90. 10.1093/gerona/glx015 PMC5861884 28475732

[43] Lamond AJ Depp CA Allison M Langer R Reichstadt J Moore DJ Measurement and predictors of resilience among community-dwelling older women. J Psychiatr Res. (2008) 43(2):148–54. 10.1016/j.jpsychires.2008.03.007 18455190 PMC2613196

[44] Zeng Y Shen K. Resilience significantly contributes to exceptional longevity. Curr Gerontol Geriatr Res. (2010) 2010(1):525693. 10.1155/2010/525693 21197075 PMC3004383

[45] Nygren B Aléx L Jonsén E Gustafson Y Norberg A Lundman B. Resilience, sense of coherence, purpose in life and self-transcendence in relation to perceived physical and mental health among the oldest old. Aging Ment Health. (2005) 9(4):354–62. 10.1080/1360500114415 16019292

[46] Henning G Lindwall M Johansson B. Continuity in well-being in the transition to retirement. GeroPsych (Bern). (2016) 29(4):225–37. 10.1024/1662-9647/a000155

[47] McLean CP Asnaani A Litz BT Hofmann SG. Gender differences in anxiety disorders: prevalence, course of illness, comorbidity and burden of illness. J Psychiatr Res. (2011) 45(8):1027–35. 10.1016/j.jpsychires.2011.03.006 21439576 PMC3135672

[48] Seedat S Scott KM Angermeyer MC Berglund P Bromet EJ Brugha TS Cross-national associations between gender and mental disorders in the world health organization world mental health surveys. Arch Gen Psychiatry. (2009) 66(7):785–95. 10.1001/archgenpsychiatry.2009.36 19581570 PMC2810067

[49] Martin LA Neighbors HW Griffith DM. The experience of symptoms of depression in men vs women. JAMA Psychiatry. (2013) 70(10):1100–6. 10.1001/jamapsychiatry.2013.1985 23986338

[50] Crowe L Butterworth P. The role of financial hardship, mastery and social support in the association between employment status and depression: results from an Australian longitudinal cohort study. BMJ Open. (2016) 6(5):e009834. 10.1136/bmjopen-2015-009834 27235296 PMC4885313

[51] McGee RE Thompson NJ. Unemployment and depression among emerging adults in 12 states, behavioral risk factor surveillance system, 2010. Prev Chronic Dis. (2015) 12:E38. 10.5888/pcd12.140451 25789499 PMC4372159

[52] Hiles SA Harvey ES McDonald VM Peters M Bardin P Reynolds PN Working while unwell: workplace impairment in people with severe asthma. Clin Exp Allergy. (2018) 48(6):650–62. 10.1111/cea.13153 29676834

[53] Alachraf K Currie C Wooten W Tumin D. Social determinants of emergency department visits in mild compared to moderate and severe asthma. Lung. (2022) 200(2):221–6. 10.1007/s00408-022-00524-3 35322286

[54] Leibel S Geng B Phipatanakul W Lee E Hartigan P. Screening social determinants of health in a multidisciplinary severe asthma clinical program. Pediatr Qual Saf. (2020) 5(5):e360. 10.1097/pq9.0000000000000360 33204931 PMC7665245

[55] Castillo EG Ijadi-Maghsoodi R Shadravan S Moore E Mensah MO Docherty M Community interventions to promote mental health and social equity. Curr Psychiatry Rep. (2019) 21(5):35. 10.1007/s11920-019-1017-0 30927093 PMC6440941

